# Return to Work After Work-Related Injuries: A Systematic Review and Meta-Analysis of Incidence and Determinants

**DOI:** 10.3390/jcm14124343

**Published:** 2025-06-18

**Authors:** Weiner Santos, Carmen Rojas, Rui Isidoro, Alejandro Lorente, Ana Dias, Gonzalo Mariscal, Ahmed Hamdy Zabady, Rafael Lorente

**Affiliations:** 1 International Doctoral School, University of Extremadura, 06006 Badajoz, Spain; weiner.santos1976@gmail.com; 2 Local Health Unit Litoral Alentejano (ULSLA), 7540-230 Santiago do Cacém, Portugal; 3 School of Industrial Engineering, University of Extremadura, 06006 Badajoz, Spain; cvrojas@unex.es; 4 University Polytechnic of Beja, Rua Pedro Suárez, Campus du University Polytechnic of Beja, 7800-295 Beja, Portugal; rui.isidoro@ipbeja.pt (R.I.); ana.dias@ipbeja.pt (A.D.); 5 Life Quality Research Centre (LQRC-CIEQV), 2001-964 Santarém, Portugal; 6 Ankle and Foot Surgery Unit, Department of Traumatology and Orthopaedic Surgery, University Hospital Ramón y Cajal, 28034 Madrid, Spain; 7 Institute for Research on Musculoskeletal Disorders, Catholic University of Valencia San Vicente Mártir, 46001 Valencia, Spain; 8 Faculty of Science, Damanhour University, Damanhour 22514, Egypt; a.zabady00711@sci.dmu.edu.eg; 9 Department of Orthopedic Surgery and Traumatology, University Hospital of Badajoz, 06010 Badajoz, Spain; rafael.lorentem@gmail.com

**Keywords:** return to work, occupational injuries, work-related injuries, rehabilitation

## Abstract

**Background:** Work-related injuries remain a considerable global burden; nevertheless, progress in occupational safety has been made in decreasing the time to return to work. This study aimed to assess the pooled incidence of Return to Work (RTW) among workers with occupational injuries and to identify the key factors influencing RTW outcomes. **Methods**: A systematic review was conducted by searching electronic databases (PubMed, Embase, Cochrane CENTRAL, Web of Science, and Scopus) to include eligible cohorts. Meta-analysis was undertaken using R software 4.5.1 with random-effects models, and heterogeneity was evaluated using the *I*^2^ statistic. **Results**: This meta-analysis included 16 cohorts, with 4164 workers. A pooled analysis of 14 studies showed that 79% of workers successfully returned to their jobs after treatment for work-related injuries (95% CI: 0.67–0.88; *p* < 0.0001; *I*^2^ = 97.5%). Meta-regression identified age as a significant moderator, with older workers showing a higher incidence of RTW. The mean time to RTW, pooled from 9 studies, was approximately 102 days, with no significant age association (*p* = 0.222). Regarding predictors, male workers had a significantly higher RTW rate than females (*p* < 0.0001). Married persons showed greater RTW rates (*p* < 0.0001). Also, workers with higher education levels were more likely to return to work (*p* = 0.0033). For injury type, lacerations were related to a greater RTW rate than crushing injuries. **Conclusions**: This meta-analysis underscored a significant overall return-to-work rate after work-related injuries, with age, sex, marital status, education level, and injury type affecting results. Advanced age and male sex were substantially correlated with increased return-to-work rates. These findings support the necessity for personalized rehabilitation programs and focused support to enhance work reintegration following occupational accidents.

## 1. Introduction

Every year, millions of workers in different industries suffer from work-related injuries, which are a serious concern in all occupational settings [1,2]. With about 340 million work-related accidents and 160 million work-related illnesses reported annually worldwide, the incidence of occupational injuries remains high despite improvements in workplace safety and technology [2]. Mechanical injuries are the primary and most-often documented injury in the industrial, construction, agricultural, and transportation sectors. They usually arise from different conditions, such as operator error, equipment malfunction, ineffective safety measures, and a failure to control machinery effectively [3].

Moreover, workers may be injured by being caught in or struck by moving machine parts, entanglement of clothing or hair, contact with sharp or abrasive surfaces, or the collapse or malfunction of heavy equipment, such as cranes and powered industrial trucks. Additional hazards encompass falls from elevations, overexertion, repetitive strain, crushing accidents, lacerations, and avulsions [3,4].

The consequences of these injuries can be serious, ranging from superficial lacerations and contusions to amputations, musculoskeletal abnormalities, or death. Specific populations, including younger and older employees and individuals in high-risk sectors, such as fishing, agriculture, and manufacturing, are particularly vulnerable [5,6,7].

Following an industrial injury, the time taken for individuals to return to work (TRTW) is a major concern with great consequences for organizational stakeholders as well as people. TRTW varies among individuals, depending on the injury severity, injury site, worker’s health condition, and the nature of the work. Extended absence combined with loss of income threatens employees’ financial stability [8,9].

The efficacy of recovery largely depends on the proficient management of the rehabilitation process. Timely intervention and tailored care plans implemented in structured rehabilitation programs have demonstrated efficacy in expediting physical recovery and reducing the likelihood of long-term disability. Integrating physiotherapy and ergonomic evaluations mitigates injury exacerbation and promotes safer reintegration into occupational environments. Enhancing employee capability typically yields improved success rates for firms that monitor progress and implement changes per healthcare providers [10,11].

TRTW schedules enhance productivity and assist in diminishing the long-term financial costs associated with recurrent absences. Multiple studies have shown that individuals re-entering the workforce via phased programs, such as part-time hours or diminished responsibilities, display superior job retention rates and faster full recovery than those returning without such provisions. Organizations gain from reduced turnover costs and retained institutional expertise, while employees attain financial security and support. A manufacturing company that adopted graduated work schedules experienced a 30% reduction in absenteeism-related costs after six months, highlighting the financial benefits of early reintegration strategies [12,13].

Older age, more severe injuries, higher pain levels, and the existence of comorbid conditions, all of which have been repeatedly linked to longer work absences, are some of the factors that influence TRTW and the incidence of RTW. On the other hand, it has been demonstrated that early graded return to work programs and greater educational attainment reduce the time spent away from work [14,15].

While multiple studies have assessed factors influencing the RTW process, the current body of evidence remains fragmented. A number of systematic reviews have examined RTW outcomes in specific populations, such as individuals with musculoskeletal disorders, low-back pain, or traumatic brain injuries, or within particular occupational sectors [16,17]. However, these reviews are typically limited in scope, focusing either on narrowly defined clinical subgroups or on intervention-specific outcomes, thereby constraining the generalizability of their findings. Therefore, the purpose of this study is to investigate how often employees who have suffered work-related injuries return to their jobs and the factors that influence this occurrence.

## 2. Materials and Methods

### 2.1. Study Design

Following the Preferred Reporting Items for Systematic Reviews and Meta-Analyses (PRISMA) guidelines, a systematic review and meta-analysis of published literature was conducted [18].

### 2.2. Search Strategy

Several electronic databases were searched, including PubMed, Embase, Cochrane Library, Web of Science (WoS), Cochrane CENTRAL, Scopus, APA PsycInfo, and Ovid. Search terms combined MeSH keywords related to “return to work,” “work-related injuries,” “occupational accident,” “industrial injury,” “vocational rehabilitation,” and “employment outcomes”.

### 2.3. Eligibility Criteria

Studies were eligible for inclusion if they involved adult participants (≥18 years) who sustained work-related injuries in occupational settings and reported outcomes related to return to work. Eligible outcomes included, but were not limited to, incidence of return to work, time to first return, and predictors of RTW, such as sex, age, educational status, type of injury, and marital status. Only observational cohort studies (prospective or retrospective) were included. Studies were excluded if they focused on non-occupational injuries, lacked quantitative return-to-work data, or were case reports, cross-sectional studies, or conference abstracts without full texts.

### 2.4. Screening Process

The records were screened twice by two separate reviewers. First, abstracts and titles were examined for appropriateness. The eligibility of full-text articles was then evaluated using the inclusion and exclusion criteria. Reviewers’ disagreements were settled by discussion.

### 2.5. Data Extraction

Using a standardized Excel form, two reviewers extracted data separately. Risk factors affecting return to work, demographic traits, and return-to-work outcomes (such as incidence and time to return) were among the extracted variables. Reviewers’ disagreements were settled by discussion.

### 2.6. Risk of Bias Assessment

The methodological quality of included studies was assessed independently by two reviewers using the National Institutes of Health (NIH) Quality Assessment Tool for Observational Cohort Studies. Any disagreements were resolved through discussion and consensus. The tool evaluated aspects such as clarity of research objectives, participant selection, exposure and outcome measures, follow-up rates, confounding control, and statistical analysis.

### 2.7. Statistical Analysis

R software 4.5.1 was used for all statistical analyses. For dichotomous outcomes, risk ratios (RRs) were computed along with 95% CIs. In single-arm studies, overall means were reported for continuous outcomes. The *I*^2^ statistic was used to evaluate heterogeneity. If significant heterogeneity was found, a random-effects model was used. Sensitivity analyses were performed to examine the strength of the results by eliminating studies with a high risk of bias or showing statistical outliers in effect size. Confounders were also used in regression analysis and subgrouping to help investigate the source of heterogeneity.

### 2.8. Ethical Considerations

Ethical approval was not required as this study utilized data from previously published literature without directly involving human participants.

## 3. Results

**Figure 1:**
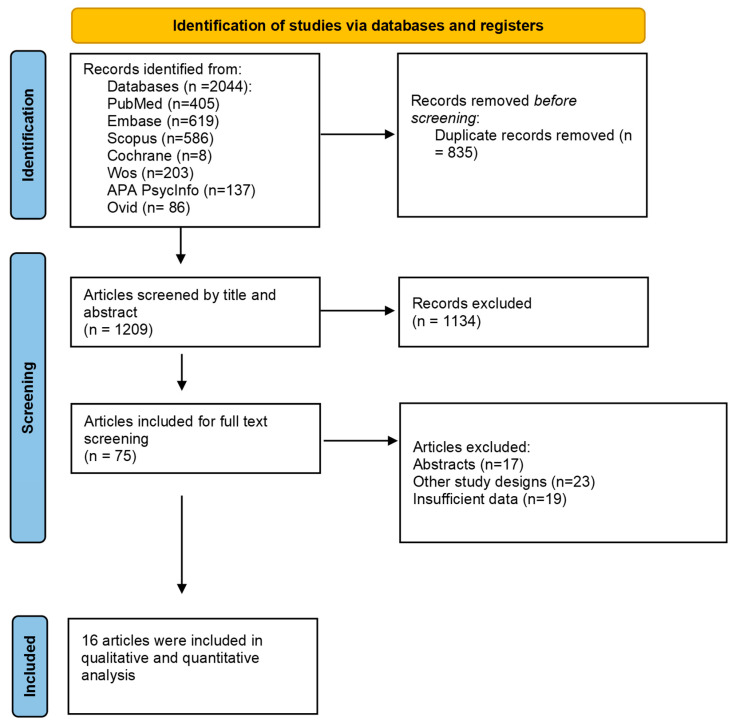
Prisma flow diagram.

A total of 2044 articles were initially retrieved from five electronic databases. Following the removal of duplicates, 1209 studies underwent title and abstract screening. Of these, 75 articles were selected for full-text assessment based on the eligibility criteria. In the end, 16 studies met the inclusion criteria and were included in the final analysis [8,9,19,20,21,22,23,24,25,26,27,28,29,30,31,32], as illustrated in Figure 1.

Out of the included 16 articles, five were prospective cohorts, while the rest were retrospective. A total of 4164 patients were included in this meta-analysis. Hand injuries were the most reported injuries among the included studies. Most of the studies were in Taiwan (37.5%). The majority were males aged between 25 and 60, as shown in Table 1.

### 3.1. Risk of Bias Assessment

Using the NIH tool, seven studies were of good quality, seven were of moderate quality, and two were of low quality, as mentioned in Table 2.

### 3.2. Outcomes

#### 3.2.1. Incidence of RTW

**Figure 2:**
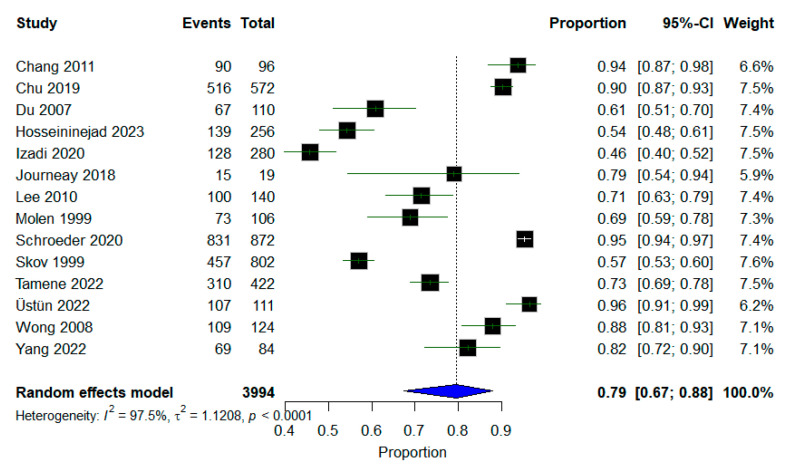
A forest plot of the incidence of workers who returned to their jobs.

A meta-analysis of fourteen studies was undertaken to explore the incidence of return to work among workers who had work-related injuries. A total of 79% of workers returned to their work after a successful treatment strategy (95% CI: 0.67–0.88, *p* < 0.0001, *I*^2^ = 97.5%). A random-effects model was employed, as shown in Figure 2.

**Figure 3:**
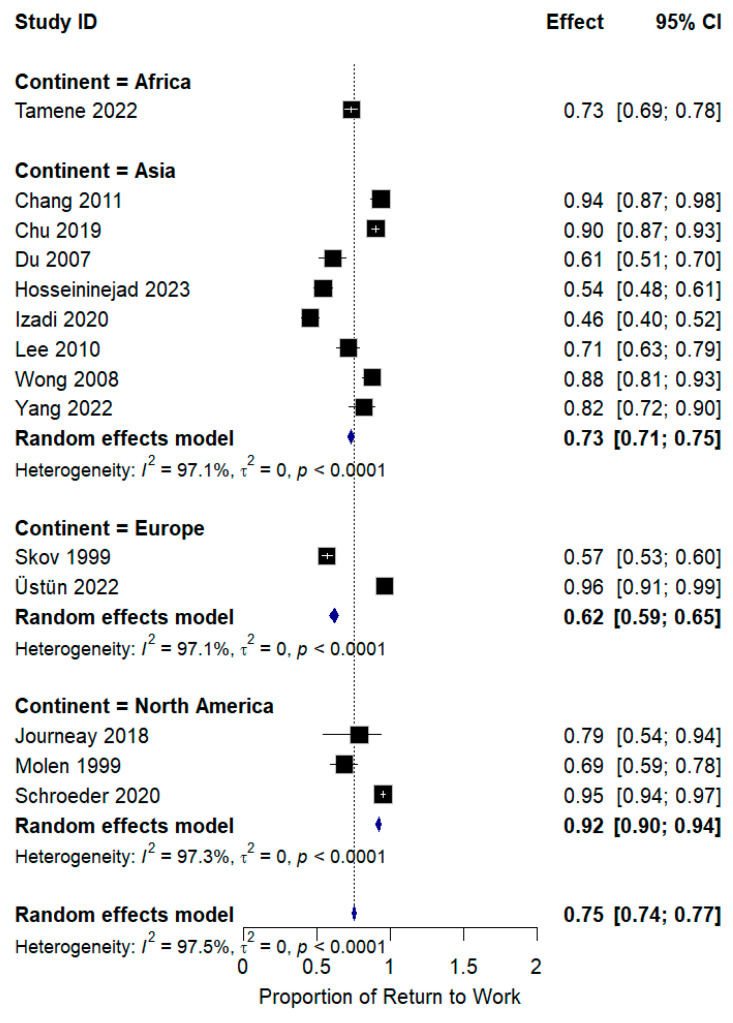
Subgroup analysis of RTW incidence according to continent.

Subgroup analysis was conducted based on the country’s continent; most of the studies were of Asian origin, but heterogeneity was high among all subgroups, with *I*^2^ equal to 97.1%. North American studies showed *I*^2^ = 97.3%, with no change in the context of results, as shown in Figure 3.

**Figure 4:**
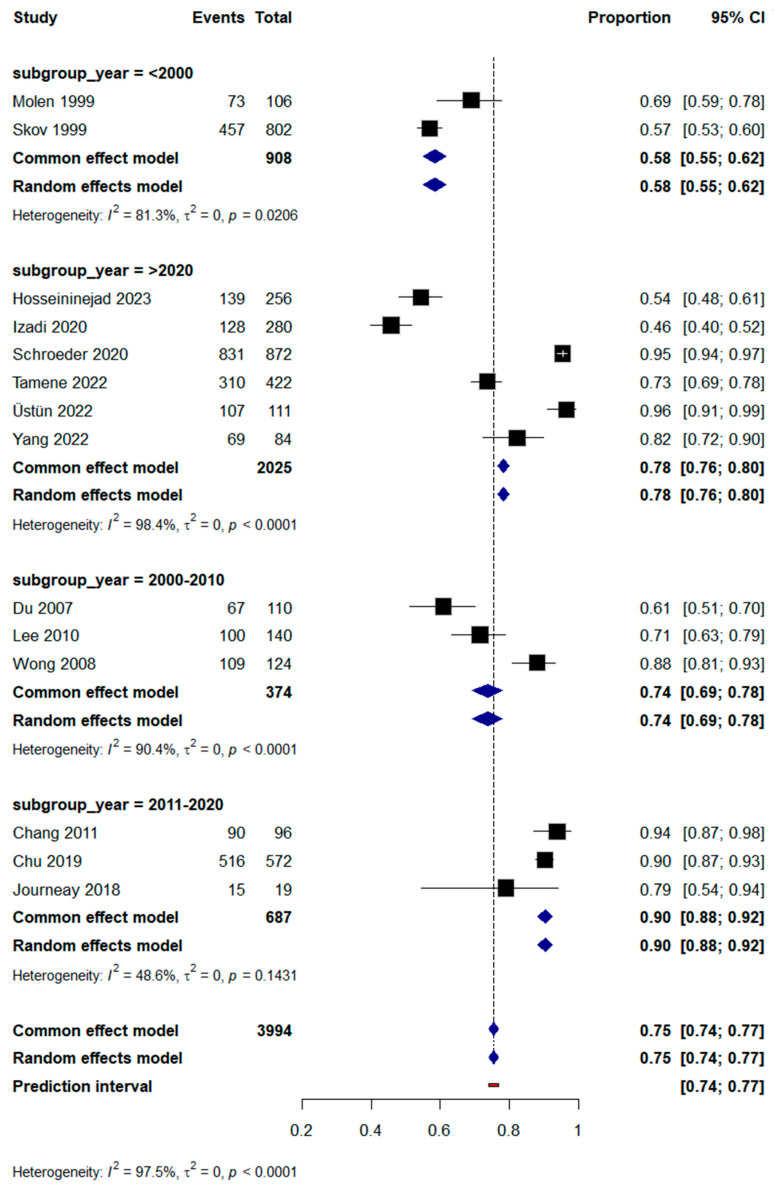
Subgroup analysis of RTW incidence based on year of publication.

Subgroup analysis was also performed based on the year of publication; we found that heterogeneity was reduced among all groups except studies published after 2020, especially for studies published between 2011 and 2020, with a significant OR of 0.90 and *I*^2^ = 48.6%, as shown in Figure 4.

#### 3.2.2. Meta-Regression

**Figure 5:**
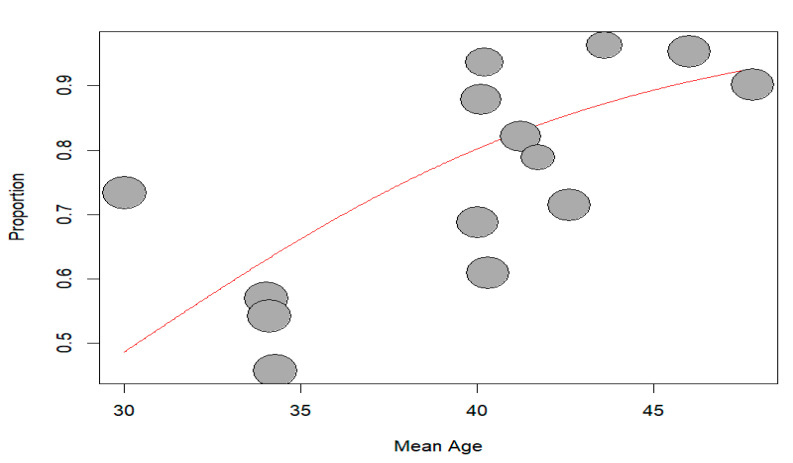
Bubble plot of meta-regression of RTW based on age.

Meta-regression based on age demonstrated that age was a statistically significant moderator of the treatment effect across studies. The regression coefficient for age was 0.1453 (SE = 0.0446), indicating that for each additional year of mean participant age, log RR increased by approximately 0.15 units. This effect was statistically significant (t = 3.26, df = 12, *p* = 0.0068), with a 95% confidence interval ranging from 0.0481 to 0.2424, suggesting a consistent and positive relationship between age and treatment effect. Older workers were associated with a higher incidence of RTW, as shown in Figure 5.

**Figure 6:**
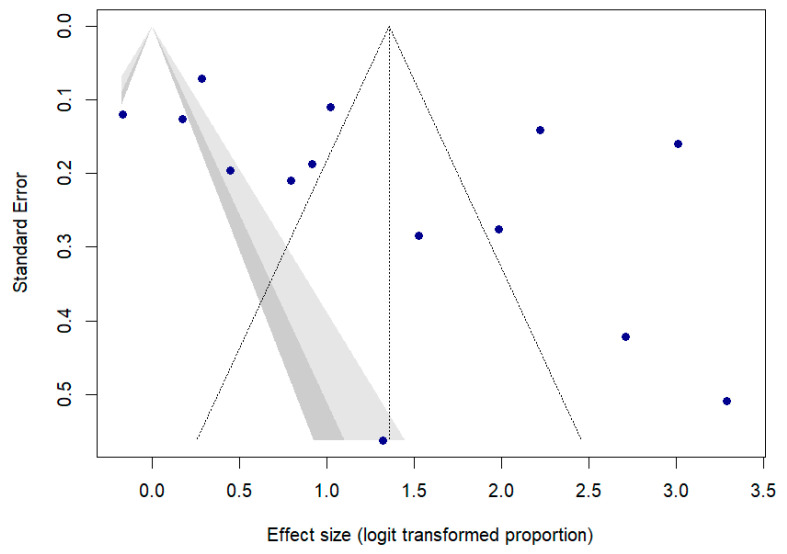
Funnel plot of return to work.

Visual inspection of the funnel plot suggested asymmetry indicative of publication bias. Egger’s test for funnel plot asymmetry was performed to assess potential publication bias in the meta-analysis. The test yielded a bias coefficient of 6.2134 (SE = 3.1211), with a *t*-value of 1.99 (df = 12) and a corresponding *p*-value of 0.0698, suggesting no strong evidence of publication bias, as shown in Figure 6.

#### 3.2.3. Time to Return to Work

**Figure 7:**
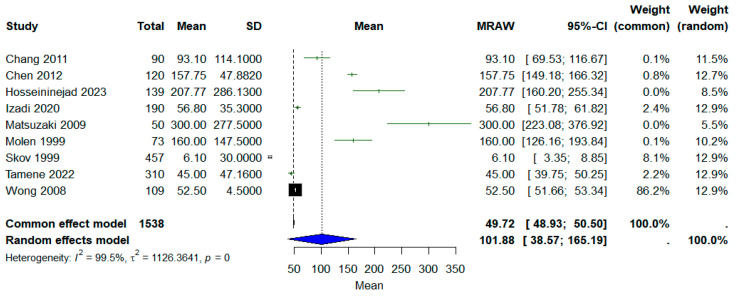
Forest plot of mean time to return to work.

Nine studies reported the mean time to return to work; a meta-analysis was conducted to calculate the overall mean TRTW. A random-effects model was employed. The pooled mean time to return to work was 101.98 days (95% CI: 38.57–165.19, *I*^2^ = 99.5%), as shown in Figure 7.

**Figure 8:**
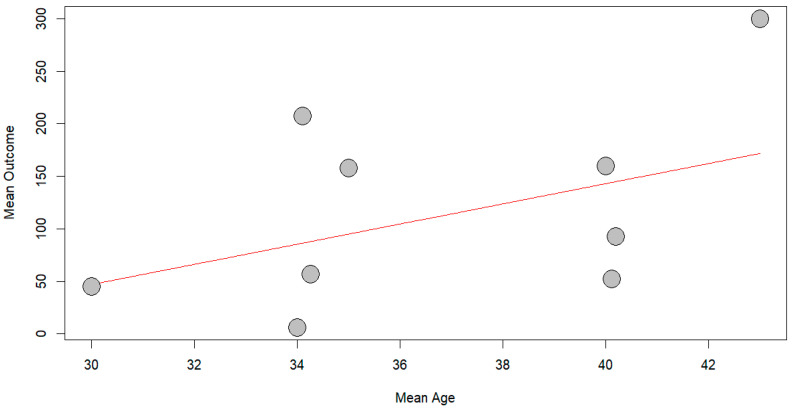
Bubble plot of meta-regression of TRTW based on age.

With respect to exploring potential heterogeneity, no significant association was observed between age and time to return to work, with *p* equal to 0.2224, as shown in Figure 8.

#### 3.2.4. Predictors of RTW

Sex:

A meta-analysis comparing the incidence of RTW between males and females showed a significantly higher likelihood of RTW among males. The pooled risk ratio was 6.12 (95% CI: 2.33–16.06, *p* < 0.0001; *I*^2^ = 95.8%). Subgroup analysis was performed based on the year of publication before and after 2015; it was found that heterogeneity was reduced among studies published before 2015, with *I*^2^ = 74.7%, while it increased among papers after 2015 to 97.2%, as summarized in Table 3.

Marital Status:

Meta-analysis revealed a significant difference between single and married workers with an RR of 0.43 (95% CI: 0.18–1.02, *p* < 0.0001; *I*^2^ = 88.3%), meaning that 57% of married workers return to their jobs. A random-effects model was used. A sensitivity analysis was performed by excluding Hosseininejad et al. [19]; heterogeneity was reduced to 46.1%, with a change in the context of the results showing no statistically significant difference between both groups, with *p* 0.1563, as summarized in Table 3.

Educational Status:

Workers with education more than grade 12 were more likely to return to work than those with less education, with a pooled RR of 0.54 (95% CI: 0.34–0.87, *p* = 0.0033; *I*^2^ = 78.1%). A sensitivity analysis excluding Tamene et al.’s study [9] showed a trend reversal, favoring lower-educated workers, with an RR of 0.47 (95% CI: 0.29–0.77, *p* = 0.2562; *I*^2^ = 26.6%). All results were obtained using a random-effects model, as summarized in Table 3.

Injury:

In terms of injury, three studies mentioned crushing and laceration injuries among workers. A meta-analysis of three studies showed that workers with lacerations had a higher incidence of RTW compared to crushing injuries, with an RR of 1.89 (95% CI: 0.11–33.74, *p* < 0.0001; *I*^2^ = 91.6%). A sensitivity analysis was performed by excluding Du et al., where heterogeneity was reduced to 19.1%, shifting results towards insignificance, supporting no difference between both groups with *p* = 0.2667, as summarized in Table 3.

## 4. Discussion

Occupational injuries are increasing nowadays, which calls for attention to explain their causes and update the incidence. This study explored the incidence and mean time to return to work.

### 4.1. Incidence and Timing of RTW

Our overall RTW incidence of 79% aligns with higher estimates for less severe or well-managed injuries. Nevertheless, the majority of injured workers eventually resumed employment, which aligns with prior evidence showing that over 60% of workers with traumatic injuries return by 6 months post-injury [33]. Several meta-analyses of return to work after injury have reported RTW rates ranging from just over 50% in major trauma populations to over 80% in specific injury cohorts. For instance, a systematic review of mild traumatic brain injury found that more than half of patients had returned by one month and over 80% by six months post-injury [17,34]. In workers with back pain, 80–90% return-to-work rates have been described for short-term absences. After major trauma, patterns and predictors of RTW revealed early sustained RTW in 51.6% of respondents and complete non-RTW in 19.7% of respondents [10]. Also, a recent meta-analysis of post-COVID-19 RTW showed a pooled RTW proportion of 60.9% at 12 weeks [35].

The persistently high RTW rates noted, even in cases of serious damage, indicate that with enough support, numerous workers can successfully reintegrate into the labor field. Timely and proactive return-to-work facilitation is essential. Several studies emphasized that multidisciplinary approaches coordinating clinical care with workplace accommodations are useful in enhancing RTW results [17]. For example, ergonomic adjustments, graded return programs, and vocational rehabilitation should be standard considerations in management plans [36].

Clinicians should, therefore, not only treat the injury but also manage pain adequately and screen for issues like depression or catastrophic thinking. Intervening on these factors, through pain management, psychosocial support, and work-focused therapy, can improve a patient’s chances of successful RTW [26].

### 4.2. Influence of Age

Generally, age has been considered a risk factor for delayed or unsuccessful RTW. Across various health conditions, older age (e.g., >50 years) is strongly associated with longer work disability and lower likelihood of RTW [11].

In contrast to numerous studies that recognize younger age as a promoter of RTW, our meta-regression revealed that older age correlated with increased RTW rates. More research is needed to study this relationship. Cancelliere et al. revealed that, across diverse injury and illness conditions, advanced age is generally associated with reduced RTW rates; however, this association is not universally consistent and may be influenced by additional factors such as employment stability, job classification, and workplace support systems [17]. Possible reasons include age-related comorbidities, slower physical recovery, and the fact that older individuals are closer to retirement. Yet our findings align with a subset of evidence suggesting a more complex reality. Some prior studies on workplace injury have noted that, when other factors are controlled, older workers can have outcomes as good as or even better than younger workers [37]. Furthermore, older employees often occupy more stable or senior positions that may be more accommodating of injury-related limitations. They may have access to light-duty roles or administrative tasks, enabling earlier return. Additionally, older workers may be especially motivated to resume work quickly, recognizing that finding new employment later in life is challenging. This heightened motivation and employer flexibility could explain why older age appeared as a favorable factor in our analysis. Socioeconomic and policy factors are also crucial in interpreting this age paradox. It is possible that many older individuals in our included studies were in higher-income or salaried positions that came with better benefits and return-to-work support. In contrast, younger injured workers, who might be overrepresented in manual labor or lower-income jobs, could face more barriers [33,38].

Our findings also indicate that younger workers may have been overrepresented in high-risk categories, while older workers with established careers benefited from resources and support that aided their return. Certain cultures or regions may have standards and regulations that promote the continued employment of older workers, so the cultural context contributes to this explanation, as most of the included studies were of Asian origin. A study of injured workers in Israel indicated that older workers were more likely to return to work in the short term, potentially due to a strong work ethic and fear of job loss [38]. However, those nearing retirement age exhibited a higher risk of exiting the workforce through early retirement. Our review indicates that the “older” workers were generally middle-aged (in their 40s or 50s) rather than at the conventional retirement age, which may partially account for the positive association observed. These individuals likely retained significant financial obligations and career commitments, motivating them to return to work. Conversely, workers approaching retirement may opt to retire following a significant injury; however, such instances may be underrepresented in the data. The finding that older age correlates with higher rates of return to work can be elucidated by various socioeconomic and cultural factors. Older workers who continue to participate in the labor force typically represent a distinct subset characterized by enhanced job security, elevated motivation, and more flexible work environments, all of which facilitate RTW [37,38]. There is a need for additional research on the impact of workplace accommodations, seniority benefits, and cultural attitudes towards older workers on successful RTW outcomes, aiming to develop effective strategies applicable to all age groups.

### 4.3. Sex Differences

Men are more likely to work than women and have a higher and faster recovery compared to women. We found that males were over six times more likely to RTW than females, with consistent results before and after 2015. Etuknwa et al. also reported inconsistent results regarding gender differences in RTW rates [39]. Conversely, findings from Rijik et al., a cohort study from the Netherlands, demonstrated no differences between males and females regarding RTW after occupational injury [40].

### 4.4. Marital Status and Education

Our findings showed that married workers initially appeared more likely to RTW, while sensitivity analysis attenuated this effect. Social support from spouses has been posited as a facilitator of RTW in chronic conditions [41], but findings have been inconsistent after sensitivity analysis, which needs further investigation in future studies. Educational attainment was positively associated with RTW, aligning with studies reporting that higher education and socioeconomic status enhance access to resources, health literacy, and workplace flexibility [42,43].

### 4.5. Impact of Injury Type

This study found that workers with lacerations had a higher RTW incidence than those with crushing injuries, echoing prognostic studies in hand trauma where injury severity inversely correlates with RTW prospects. Crushing injuries often entail more extensive soft-tissue and neurovascular compromise, prolonging recovery and complicating return. This aligns with prior prognostic studies in hand trauma and the general injury literature, where the severity and complexity of injury inversely correlate with RTW prospects [44].

### 4.6. Strengths and Limitations

Although this study provides a comprehensive assessment of RTW, several limitations should be mentioned. First, most of our analyses had high heterogeneity (*I*^2^ > 90%), reducing confidence in the pooled estimates. The predominance of retrospective cohort studies also indicates a potential selection and information bias. Moreover, there was a significant publication bias among the included studies, as suggested by funnel plot asymmetry, which further limits the reliability of the findings. Additionally, the geographical concentration of studies in Asia and the lack of representation from low-resource settings constrain the external validity and generalizability of the results. Furthermore, the sensitivity analyses caused a shift in results, which need to be studied further in future studies.

Future research should focus on establishing standardized metrics for RTW, including clear distinctions between sustainable and initial RTW, alongside consistent follow-up durations. Large-scale, prospective multicenter cohort studies are recommended to mitigate bias and allow for the evaluation of time-varying predictors. Moreover, incorporating psychosocial variables, such as self-efficacy, workplace support, and mental health, may clarify the non-clinical determinants of RTW. Intervention trials assessing the efficacy of targeted RTW programs, including workplace accommodations and graded return protocols, using randomized designs, are also needed. Finally, expanding research to include underrepresented regions and occupational sectors would enhance the global applicability of findings.

This review highlighted the need for improved research quality in occupational injury rehabilitation, focusing on potential directions for future studies. We suggest using prospective designs with thorough follow-up to generate more dependable prognostic evidence. Standardization of outcome measurement is crucial, and understanding the long-term employment pathways of injured workers is essential. Comparative studies across different countries or labor systems can elucidate the impact of policies on return-to-work rates. Qualitative research can offer insights into personal, cultural, and organizational factors affecting return to work, and addressing modifiable factors is essential. Employer training and workplace accommodation programs can enhance return-to-work outcomes. Addressing these gaps will enhance guidance for clinicians and policymakers.

## 5. Conclusions

This meta-analysis demonstrated that approximately 80% of injured workers return to work within an average of 102 days. Factors such as older age, male gender, and higher levels of education were associated with more favorable RTW incidence. Implementing individualized rehabilitation strategies and supportive workplace policies remains essential to improve RTW outcomes across various injury types and worker populations, so further studies are warranted to explore these points.

## Figures and Tables

**Table 1:** Baseline characteristics and quality assessment of included studies.

Study ID	Study Design	Country	Injury Site	Treatment	Period of Data Collection	Age Means (SD)	Sex M/F
Skov et al., 1999 [8]	Retrospective cohort	Denmark	Hand injury	Surgical, clinical, and rehabilitation	-		
Tamene et al., 2022 [9]	Retrospective cohort	Ethiopia	Spine (29.6%), the upper limbs (34.1%), head and neck (21.1%), and the lower limbs (15.2%).	Surgical, clinical, and rehabilitation	Between 1 January 2017, and 31 December 2021	30 (4.5)	398/24
Hosseininejad et al., 2023 [19]	Retrospective cohort	Iran	Upper limb injuries	Physiotherapy and prosthesis use	March 2011 to December 2018	34.10 ± 9.04	245/11
Chen et al., 2012 [20]	Prospective cohort	Taiwan	Hand injury	Surgical, clinical, and rehabilitation	-	35 (6.7)	92/28
Chang et al., 2011 [21]	Retrospective cohort	Taiwan	Traumatic hand injury	-	From 2003 to 2006	40.2 (12.5)	80/16
Molen et al., 1999 [22]	Prospective cohort	USA	Hand injury	-	-	-	-
Wong, 2008 [23]	Prospective cohort	China	Hand Injury	Surgical reconstruction	-	40 (12)	100/24
Matsuzaki, 2009 [24]	Retrospective cohort	Japan	Hand Injury	Surgical, clinical, and rehabilitation	Between October 1990 and August 2003	43 (18–69)	40/10
Journeay et al., 2018 [25]	Retrospective cohort	Canada	Lower extremity	Surgical amputation	-	41.7 (15)	19/0
Yang et al., 2022 [26]	Retrospective Cohort	Taiwan	The upper limbs (61.9%), the lower limbs (13.1%), others (25%)	-	September 2016 and December 2018	41.24 ± 12.33	54/30
Lee et al., 2010 [27]	Retrospective cohort	Taiwan	Hand injury	Surgical, clinical, and rehabilitation	Between September and November 2008	42.6 (12.9)	99/41
Schroeder et al., 2020 [28]	Retrospective cohort	Canada	Upper limb injuries	Physiotherapy, pain treatment, surgery, psychological treatment	Between April 2011 and September 2015	46 (11)	570/298
Üstün et al., 2022 [29]	Retrospective cohort	Turkey	Hand injury	Surgical, clinical, and rehabilitation	Between April 2020 and October 2021	43.6	99/12
Izadi et al., 2020 [30]	Prospective cohort	Iran	Hand injury	-	Between July 2017 and February 2018	34.26 (9.67)	271/8
Chu et al., 2019 [31]	Prospective cohort	Taiwan	-	Physiotherapy, pain treatment, pharmacist assessment, surgery, psychology	Between 1 February and 31 August 2009.	47.8 (11.1)	386/186
Du et al., 2007 [32]	Retrospective cohort	Taiwan	Upper limb injuries	Surgical, clinical, and rehabilitation	16 August to 15 October 2003	40.3 ± 9.8	91/19

**Table 2:** Quality assessment of included studies using NIH tool.

Study	Population Defined	Exposure Measured	Outcome Measured	Confounding Controlled	Follow-Up Adequate	Overall Rating
**Skov et al., 1999 [8]**	Yes	Yes	Yes	No	Yes	High
**Tamene et al., 2022 [9]**	Yes	Yes	Yes	Yes	Yes	High
**Hosseininejad et al., 2023 [19]**	Yes	Yes	Yes	Yes	Yes	Moderate
**Chen et al., 2012 [20]**	Yes	Yes	Yes	Yes	Yes	Moderate
**Chang et al., 2011 [21]**	Yes	Yes	Yes	Yes	Yes	Moderate
**Molen et al., 1999 [22]**	Yes	Yes	Yes	No	No	Low
**Wong, 2008 [23]**	Yes	Yes	Yes	Yes	Yes	Moderate
**Matsuzaki, 2009 [24]**	Yes	Yes	Yes	Yes	Yes	High
**Journeay et al., 2018 [25]**	Yes	Yes	Yes	Yes	Yes	High
**Yang et al., 2022 [26]**	Yes	Yes	Yes	Yes	Yes	Moderate
**Lee et al., 2010 [27]**	Yes	Yes	Yes	Yes	Yes	High
**Schroeder et al., 2020 [28]**	Yes	Yes	Yes	Yes	Yes	High
**Üstün et al., 2022 [29]**	Yes	Yes	Yes	Yes	Yes	High
**Izadi et al., 2020 [30]**	Yes	Yes	Yes	Yes	Yes	Moderate
**Chu et al., 2019 [31]**	Yes	Yes	Yes	Yes	Yes	Moderate
**Du et al., 2007 [32]**	Yes	Yes	Yes	No	No	Low

**Table 3:** Summary of meta-analysis for predictors of return to work (RTW).

Predictor	Comparison	Included Studies	Risk Ratio (RR)	95% Confidence Interval	*p*-Value	*I*^2^ (%)	Model	Interpretation
Sex	Male vs. Female	Chang et al., 2011 [21], Du et al., 2007 [32], Hosseininejad et al., 2023 [19], Izadi et al., 2020 [30], Lee et al., 2010 [27], Schroeder et al., 2020 [28], Tamene, 2022 [9], and Yang et al., 2022 [26]	6.12	2.33–16.06	<0.0001	95.8	Random-effects	Males had significantly higher RTW rates.
Sex subgrouping	Subgroup: Before 2015	Chang et al., 2011 [21], Du et al., 2007 [32], and Lee et al., 2010 [27]	4.25	1.38–13.05	0.0193	74.7	Random-effects	Moderate heterogeneity in older studies, with no change in the context of results
	Subgroup: 2015 and after	Hosseininejad et al., 2023 [19], Izadi et al., 2020 [30], Schroeder et al., 2020 [28], Tamene, 2022 [9], and Yang et al., 2022 [26]	7.95	1.29–49.00	<0.0001	97.2	Random-effects	Very high heterogeneity in recent studies, with no change in the context of results
	Subgroup difference test		—	—	0.3750	—	—	No statistically significant subgroup difference (χ^2^ = 0.79, df = 1)
Marital Status	Married vs. Single	Hosseininejad et al., 2023 [19], Du et al., 2007 [32], Lee et al., 2010 [27], and Yang et al., 2022 [26]	0.43	0.18–1.02	<0.0001	88.3	Random-effects	Married workers showed higher RTW
Marital Status (SA *)	Married vs. Single (SA)	Sensitivity analysis excluding Hosseininejad et al. [19]	0.55	0.30–1.01	0.1563	46.1%	Random-effects	There was no difference between the two groups in terms of RTW incidence.
Education	>Grade 12 vs. ≤Grade 12	Hosseininejad et al., 2023 [19], Tamene, 2022 [9], Lee et al., 2010 [27], and Yang et al., 2022 [26]	0.54	0.34–0.87	0.0033	78.1	Random-effects	Higher education is linked to more RTW.
Education (SA *)	>Grade 12 vs. ≤Grade 12	Sensitivity analysis excluding Tamene et al. [9]	0.47	0.29–0.77	0.2562	26.6	Random-effects	There was no difference between the two groups in terms of RTW incidence.
Injury	Laceration vs. Crushing	Üstün et al., 2022 [29], Du et al., 2007 [32], and Wong, 2008 [23]	1.89	0.11–33.74	<0.0001	91.6	Random-effects	Workers with lacerations had higher RTW
Injury (SA *)	Laceration vs. Crushing	Sensitivity analysis excluding Du et al. [32]	3.99	0.20–80.10	0.2667	19.3	Random-effects	There was no difference between the two groups in terms of RTW incidence.

* SA: Sensitivity Analysis.

## Data Availability

Primary data for the systematic literature review are reported in the referenced publications.

## Abbreviations

The following abbreviations are used in this manuscript:

CI	Confidence Interval
PRISMA	Preferred Reporting Items for Systematic Reviews and Meta-Analyses
RTW	Return to Work
